# White Adipose Tissue Resilience to Insulin Deprivation and Replacement

**DOI:** 10.1371/journal.pone.0106214

**Published:** 2014-08-29

**Authors:** Lilas Hadji, Emmanuelle Berger, Hédi Soula, Hubert Vidal, Alain Géloën

**Affiliations:** 1 Université de Lyon, CARMEN INSERM U1060, INSA-Lyon, F-69621, Villeurbanne, France; 2 IXXI Complex Systems Institute, F-69007, Lyon, France; 3 EPI BEAGLE INRIA F-69621, Villeurbanne, France; University of Pécs Medical School, Hungary

## Abstract

**Introduction:**

Adipocyte size and body fat distribution are strongly linked to the metabolic complications of obesity. The aim of the present study was to test the plasticity of white adipose tissue in response to insulin deprivation and replacement. We have characterized the changes of adipose cell size repartition and gene expressions in type 1 diabetes Sprague-Dawley rats and type 1 diabetic supplemented with insulin.

**Methods:**

Using streptozotocin (STZ)-induced diabetes, we induced rapid changes in rat adipose tissue weights to study the changes in the distribution of adipose cell sizes in retroperitoneal (rWAT), epididymal (eWAT) and subcutaneous adipose tissues (scWAT). Adipose tissue weights of type 1 diabetic rats were then rapidly restored by insulin supplementation. Cell size distributions were analyzed using multisizer IV (Beckman Coulter). Cell size changes were correlated to transcriptional regulation of genes coding for proteins involved in lipid and glucose metabolisms and adipocytokines.

**Results:**

The initial body weight of the rats was 465±5.2 g. Insulin privation was stopped when rats lost 100 g which induced reductions in fat mass of 68% for rWAT, 42% for eWAT and 59% for scWAT corresponding to decreased mode cell diameters by 31.1%, 20%, 25.3%, respectively. The most affected size distribution by insulin deprivation was observed in rWAT. The bimodal distribution of adipose cell sizes disappeared in response to insulin deprivation in rWAT and scWAT. The most important observation is that cell size distribution returned close to control values in response to insulin treatment. mRNAs coding for adiponectin, leptin and apelin were more stimulated in scWAT compared to other depots in diabetic plus insulin group.

**Conclusion:**

Fat depots have specific responses to insulin deprivation and supplementation. The results show that insulin is a major determinant of bimodal cell repartition in adipose tissues.

## Introduction

Changes in adipose mass throughout life are a common phenomenon in most individuals. Nevertheless nowadays the prevalence of individuals with an excessive accumulation of adipose tissue (obesity) has reached epidemic proportions. The main problem is that obesity is associated with metabolic disorders including insulin resistance, type 2 diabetes, dyslipidemia, cardiovascular diseases and cancers, resulting in a decreased lifespan [1]. White adipose tissue is recognized as a dynamic endocrine organ able to produce and release numerous bioactive polypeptides known as adipokines. Obesity is defined as an excessive growth of adipose tissue resulting from increased number and size of adipose cells. As such, it is likely that adipokines could play an important role in the development of diseases related to obesity [2]. Furthermore, it is now well known that distribution of body fat plays a critical role in the metabolic complications related to obesity [3], [4]. Besides the importance of body fat distribution, evidence increasingly shows a significant role of adipose cell size [5], [6]. Adipose tissue of obese animals and humans is infiltrated by macrophage population. That infiltration is correlated to adipocyte size [7]. Numerous studies have described a bimodal cell size distribution in adipose tissue [8], [9], [10] allowing to classify adipocytes into small (below the nadir) and large sizes. These studies suggested that metabolic properties, such as insulin sensitivity and secretion of adipokines, depend on cell size distribution rather than mean cell size. Although such studies suggest that the development of metabolic deseases is conditionned not only by the quantity but also by the quality of adipose tissue (i.e. cellularity), very little is known about the dynamic of changes of adipose tissue cellularity. Differences in adipose cell size contribute to the health risks of obesity through altered production of hormones such as adiponectin and leptin [11]. Adipocyte size is an important determinant of adipokine secretion [5], [12], [13]. Indeed, enlarged adipocytes are associated with metabolic abnormalities such hyperinsulinemia, glucose intolerance, dyslipidemia [14]. Skurk et al. [15] observed a differential expression of pro- and anti-inflammatory factors with increased adipocyte size resulting in a shift toward dominance of pro-inflammatory adipokines largely as a result of a dysregulation of very large cells. More recent results showed an association between small adipose cells and inflammation [16]. Although it is assumed that insulin is a positive regulator of fat cell development, little is known about its role on adipose cell size regulation [17].

The aim of the present study was to investigate the role of insulin on adipose tissue plasticity through the changes of adipose cell sizes. For that purpose, we induced rapid changes in adipose tissue weight to study the changes in the distribution of adipose cell sizes. Adipose cell size is determined by the equilibrium between lipogenesis and lipolysis. Lipolysis is mainly triggered by the sympathetic system for the lipolysis itself and by insulin as a strong antilipolytic agent while lipogenesis is mainly controled by insulin [18], [19]. Insulin deprivation (type 1 diabetes) results in a fast and marked loss of adipose tissue mass that can be rapidly restored by insulin supplementation, resulting in hypertrophia and hyperplasia of white adipose tissue (WAT) [20], [21]. Since adipose tissue distribution and function in different body compartments can be heterogeneous, we measured cell size distribution in different fat depots (epididymal, retroperitoneal and subcutaneous) [22]. We also studied the relationship between cell size distribution and the transcriptional regulation of mRNAs encoding for proteins involved in metabolism, adipogenesis, or hormonal functions. Our results show that insulin is a master trigger of the bimodal repartition of adipose cell size distribution. Adipose tissue is strongly resilient since it can lose 50% of its mass, lose its bimodal cell size distribution during insulin deprivation and recover both parameters in response to insulin supplementation.

## Methods

### Animals and type 1 diabetes induction

Adult male Sprague-Dawley rats (Harlan Gannat, France) were housed in an air-conditioned room with a controlled environment (24±1°C) under a 12 h light and dark cycle with free access to food [3.2 kcal/kg, 65% carbonhydrates, 11% lipids, 24% proteins (wt/wt), AO3, SAFE, Augy, France] and tap water. All experiments were performed according to the guidelines issued by the French Ministère de l'Agriculture (n°87-848) and the European Union Council Directive for the Care and Use for the Laboratory Animals of November 24^th^, 1986 (86/609/EEC). The experimental protocol was approved by the local ethics committee on animal experimentation (Comité Ethique de l'expérimentation animale de l'INSA de Lyon, Cetil, n° 022012CETIL). Twelve week-old rats weighing between 300–350 g were randomly divided into three groups: control (n = 7), diabetic (n = 10) and diabetic treated with insulin (n = 10) rats. The type 1 diabetes was induced by a single intraperitoneal injection of streptozotocin (STZ, 45 mg/kg) (Sigma, St Quentin Fallavier, France). Untreated diabetic rats were euthanized after the animals lost 100 g of body mass. A second group of diabetic rats was treated with insulin (8.5 U.kg^−1^.day^−1^), they were euthanized when their body mass reached that of controls at baseline. Rats were euthanized by a lethal injection of pentobarbital.

### Isolation of adipocytes

Adipocytes were isolated from 2.0 g of epididymal fat by a modification of Rodbell's original procedure [23]. Adipose tissues were weighed, minced and digested in a 20-mL polyethylene vial containing 8 mL of Krebs Ringer Bicarbonate (KRB) buffer with 1 mg/mL collagenase (type 2, *Clostridium histolyticum*), 1% BSA (fraction 5, fatty acid free) and 200 nM adenosine (Sigma). The vial was shaken at 80 cycles/min at 37°C. The resulting cell suspension was filtered through a nylon mesh (200 µm) and washed three times with 5 mL of fresh KRB buffer and 1% BSA and two times with the same buffer containing 4% BSA. Then, adipocytes were resuspended in KRB buffer and 4% BSA. A sample of the final cellular suspension was counted in a hemocytometer after staining with trypan blue.

### Blood samples and tissues dissections

Blood was rapidly withdrawn by cardiac puncture and collected in heparinized tubes, centrifuged 5 min at 4000 *g* to prepare plasma. Plasma samples were collected, frozen in liquid nitrogen and stored at −80°C until analysis. White adipose tissues (WAT) from three different anatomical depots: epididymal (eWAT), retroperitoneal (rWAT) and subcutaneaous (scWAT) were dissected out according to anatomical landmarks and weighted. One sample of each tissue was fixed in osmium tetroxide for the measure of cellularity, another sample was snap frozen in liquid nitrogen and stored at −80°C.

### Cellularity study: Measurement of adipocyte size and number

The method for measuring adipose cell size was a slight modification of Hirsh and Gallian [24]. Briefly, 50–90 mg tissue samples from each depot were rinsed twice with NaCl 0,9% (w/v) at 37°C, twice in 50 mM 2,4,6-trimethylpyridine/NaCl 0,9% and fixed in 0,12 M osmium tetroxide/50 mM 2,4,6-trimethylpyridine/NaCl 0,9% (Sigma Aldrich, Saint Quentin Fallavier, France) for 96 hours at room temperature. Samples were washed with saline for 24 h, then the saline was replaced by 10 mL of 8 M urea for 72 h with occasional swirling to liberate the cells. Samples were then washed with 0.01% TRITON X-100 (v/v) in NaCl buffer. Before analysis, the supernatant was discarded and cells were resuspended in glycerol. Cells were then diluted. The adipocyte size distribution was determined using a Beckman Coulter Counter Multisizer IV with a 400 µm aperture. The range of cell sizes that can effectively be measured using this aperture is 20–240 µm. After collection of pulse sizes the data were expressed as particle diameters and displayed as histograms of counts against diameter using linear bings and linear scale for the cell diameters. Cell size distributions were drawn from measurement of at least 21,000 cell diameters per animal.

As noted before, adipose cell size repartition is not homogeneous but bimodal [8], [9], [10]. Two populations of cells can be distinguished according to their size. A cell population having small size and a cell population with much larger size. These two populations are separated by the nadir, which is the size at which the cell frequency is the lowest. The mode is the size at which most cell size of the largest population is observed. Finally the width refers to the width of the gaussian curve at half the maximum drawn from the frequency of diameters of the larger cell population. To obtain indications on the cell size distribution like mode, width and nadir, cellularity histograms were fitted against a sum of two exponentials (to describe small adipocytes) and a gaussian (for bigger adipocytes) [25]. A simple Levenberg-Marquardt algorithm was used to minimize quadratic error. 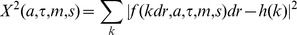
with *k* being the bin index, *dr* the bin width and



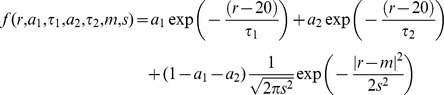



The mode of the distribution refers to the parameter m and the width as s. The nadir of the distribution is found numerically by looking for the root of the derivative of the fit.

From cell size distribution using a Coulter Counter, we estimated volume-weighted mean cell size and total cell number of the different adipose depots according to Jo et al. [26].

### Plasma parameters

Triglycerides and cholesterol were assayed using the following commercially available kits, Triglycerides PAP (BioMérieux, Marcy l'Etoile, France) Cholesterol RTU (BioMérieux) according to the manufacturer's recommendations. Plasma leptin, adiponectin and insulin concentrations were measured by means of ELISA tests (SpiBio, Montigny Le Bretonneux, France and Eurobio Courtabœuf, France).

### mRNA quantification by qRT PCR

Total RNA purifications were performed on either 5.10^5^ isolated adipocytes or 100 mg of frozen tissue according to standard protocol (Lipid Quick prep mRNA, Qiagen, Courtaboeuf, France) including a DNase treatment. RNA integrity was assessed with the Agilent 2100 Bioanalyzer and RNA 6000 LabChip Kit (Agilent Technologies, Massy, France). First strand cDNAs were synthesized from 1 µg of total RNA in the presence of 100 U of Superscript II (Invitrogen-Life Technologies, Eragny, France) and random hexamers and oligo(dT) primers (Promega, Charbonnières, France). Real-time quantitative PCR was performed using ABsolute QPCR SYBR Green ROX Mix (Abgene, Courtaboeuf, France) with a Rotor-GeneTM 6000 system (Corbett Life Science, Cambridgeshire, UK).

Gene names, references, primers and respective qPCR conditions are reported in **Table 1**. Fold changes stimulated versus control were normalized to hypoxanthine phosphoribosyltransferase (HPRT). All quantifications were performed in duplicates on fat tissues from three animals and data are presented as mean normalized fold change values ± standard deviation.

**Table 1:** Primers used for qRT PCR analysis of rat gene expression.

Acronym	Name	Primer sequences (5′-3′) or references	Tm (°C)
ADIQ	Adiponectin	Romero et al, 2009	55
ADRß3	Adrenergic receptor béta 3	Barendrecht et al, 2009	60
APELIN	Apelin	For-CTGCTCTGGCTCTCCTTGA	60
		Rev-TGGTCCAGTCCTCGAAGTTCT	
CEBPα	CCAAT/enhancer binding protein (C/EBP), alpha	For-TGCCCATGGCCTTGACCAAGGAG	60
		Rev-GCAAGGCCAAGAAGTCGGTGGAC	
COL6A3	Collagen, type VI, alpha 3	For_CATTCACTTCACGGATGGAG	60
		Rev_AGGTTAGCCACACGTTCAAG	
FASN	Fatty acid synthase	For-GGTTTGGAATGCTGTCCAGGG	60
		Rev-GTGCACCCCATTGAAGGTTCC	
FN1	Fibronectin 1	For_GCTCTCTCTCAGACAACCATC	60
		Rev_CAGAGTCGCACTGGTAGAAG	
GLUT4(SLC2A4)	Solute carrier family 2 (facilitated glucose transporter), member 4	For-CTGGGTTTCACCTCCTGCTC	60
		Rev-GGGTTTCCAGTATGTTGCGG	
HSL(LIPE)	Hormono-Sensitive Lipase	Deng et al, 2006	60
HPRT	Hypoxanthine phosphoribosyltransferase 1 (Lesch-Nyhan syndrome)	For-AGTTGAGAGATCATCTCCAC	55
		Rev-TTGCTGACCTGCTGGATTAC	
IL6	Interleukin-6	Romero et al, 2009	60
LEPTIN	Leptin	For-TGCCAGTGTCTGGTCCATCTTG	60
		Rev-CACCAGGATCAATGACATTTC	
LPL	Lipoprotein Lipase	Somm et al, 2009	60
MCP1(CCL2)	chemokine (C-C motif) ligand 2	Romero et al, 2009	55
PAI1	Serpine1	Romero et al, 2009	60
Pbef1	Visfatin	Romero et al, 2009	60
PPARγ2	Peroxisome proliferator-activated receptor gamma	For-CTGACCCAATGGTTGCTGATTAC	60
		Rev-GGACGCAGGCTCTACTTTGATC	
SREBP1c	Sterol regulatory element binding transcription factor 1c	Somm et al, 2009	60
TBP	TATA box binding protein	For-TTCACATCACAGCTCCCCAC	
		Rev-TGGTGTGCACAGGAGCCAAG	
TNFα	Tumor Necrosis Factor	Romero et al, 2009	60

### Statistical analysis

Data are expressed as means ± standard error of the mean or standard deviation. Statistical analyses were performed with StatView 4.5 software for Windows, the data were analyzed using one way ANOVA following by Fisher's protected least significance difference (PLSD), *post hoc* test. Statistical significance was accepted at p<0.05.

## Results

### Body mass

**Figure 1:**
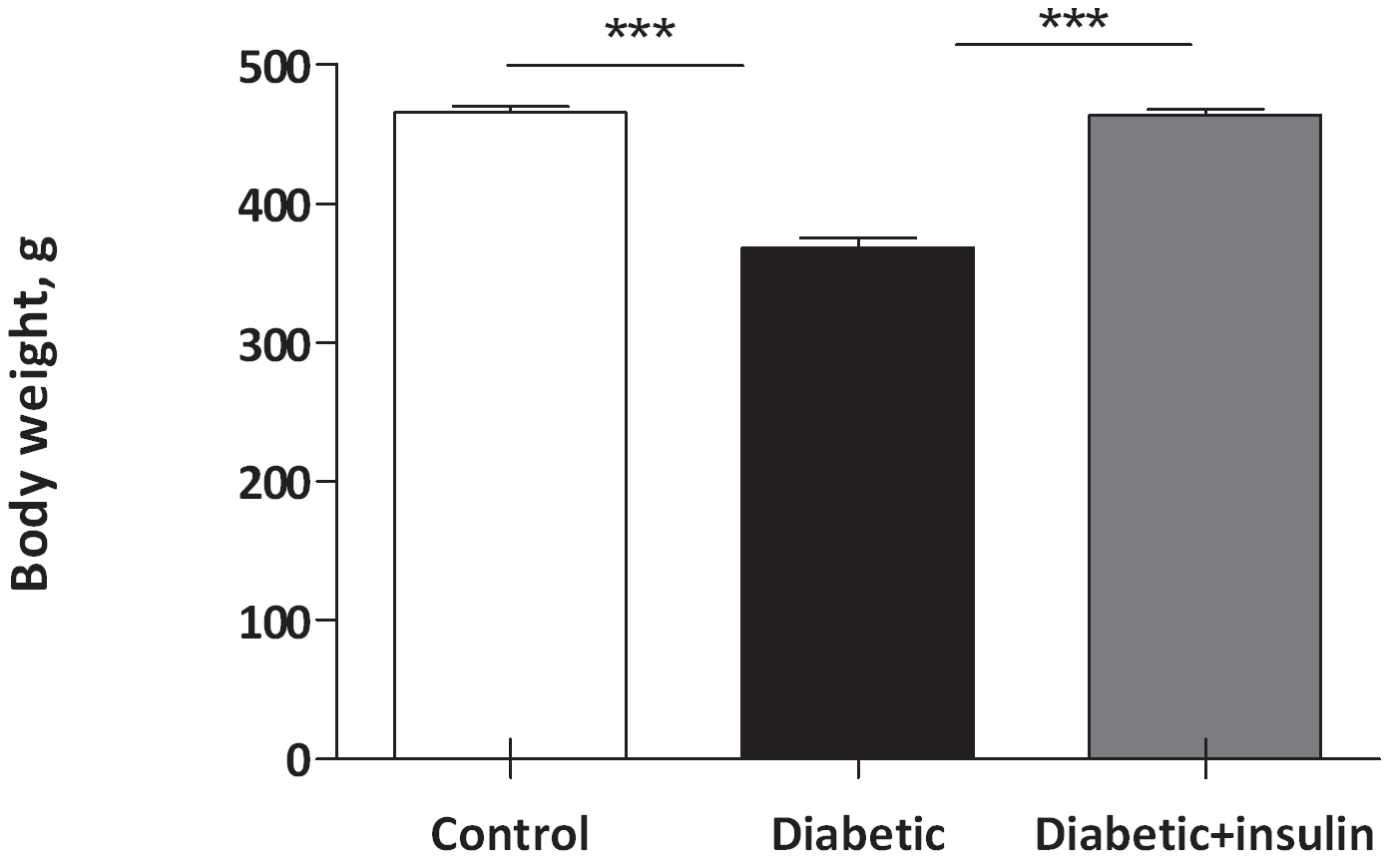
Effect of insulin privation on body weight during 10 days. The diabetic group has lost 100 g of its initial mass. After 10 days of insulin supplementation, the diabetic group has recovered its initial mass. In each group values are mean ±SEM. ANOVA, *** p<0.001.

Initial body mass of the animals was 465±5 g. Ten days of type 1 diabetes induced a reduction of 21% of the initial body mass (368.6±7.9 g, p<0.001) (**Figure 1**). Insulin replacement resulted in a rapid increase in body mass. The mean body mass of diabetic insulin treated rats rapidly reached the value of control rats (462.0±6.7 g *versus* 464.1±8.4 g, not significant).

### Plasma parameters

Blood metabolic parameters for the three groups are shown in **Table 2**. Plasma insulin concentration was markedly decreased by streptozotocin and increased by supplementation. A 4-fold increase in triglyceride concentrations was observed in the diabetic group compared with the control group, whereas after insulin treatment that value approximated the initial concentration. Cholesterol concentration remained unchanged during diabetes but increased after insulin replacement. Leptin, the protein encoded by *ob* gene, is known to regulate apetite and energy expenditure. A 3-fold decrease in leptin concentration was observed in diabetic group *versus* control. After insulin replacement, the concentration of leptin increased 3 times compared with the control group. The concentration of plasmatic adiponectin was not affected by diabetes, but increased after insulin replacement.

**Table 2:** Plasma parameters in control, diabetic and diabetic treated with insulin rats.

	Control	Diabetic	Diabetic plus insulin
Glucose mg/mL	112.4±4.4^a^	526±24^b^	56.7±4.54^a^
Triacylglycerol mM	0.86±0.12^a^	3.61±0.4^b^	0.53±0.06^a^
Total Cholesterol mM	3.08±0.06^a^	3.11±0.19^a^	4.05±0.06^b^
Plasma insulin ng/mL	1.5±0.4^b^	0.28±0.02^a^	13.9±1.3^c^
Leptin ng/mL	6.36±0.84^a^	2.06±0.21^b^	19.21±1.84^c^
Adiponectin µg/mL	5.04±0.09^a^	5.00±0.33^a^	7.57±0.13^b^

All results are mean ±SEM. Significant differences between controls (n = 7), diabetic (n = 10) and diabetic treated with insulin (n = 10) rats are estimated by ANOVA (a, b, c, P<0.05).

### Regional fat mass reduction induced by diabetes

All adipose tissue weights were significantly reduced in the diabetic group, nevertheless each fat depot was differentially affected by the deprivation in insulin (**Table 3**). The mass of eWAT showed a reduction of 42% compared with the control. After insulin replacement, eWAT mass was restored. In diabetic rats, rWAT mass showed a reduction of 68% compared with the control. Insulin supplementation failed to bring rWAT mass back to control values. scWAT showed a reduction of 59% compared with control. After insulin treatment, scWAT mass was higher than the one of control.

**Table 3:** Comparison of adipose cell size variables and number in eWAT, rWAT and scWAT in each condition.

	Control	Diabetic	Diabetic plus insulin
	(n = 4–6)	(n = 10)	(n = 10)
**eWAT**			
Mass (g)	4.7±0.1^a^	2.7±0.3^b^	4.5±0.3^a^
Mean (µm)	78.5±4.3^ab^	72.0±2.9^a^	83.7±2.6^b^
Mode (µm)	98.6±3.9^a^	79.0±4.7^b^	99.7±3.2^a^
Nadir (µm)	52.1±6.5^a^	42.6±4.0^b^	64.3±2.6^c^
Width (µm)	12.7±1.3	14.9±1.6	12.2±0.4
Cell number (10^6^)	11.5±1.2	11.8±1.7	11.4±0.7
**rWAT**			
Mass (g)	4.1±0.2^a^	1.3±0.3^b^	2.9±0.2^c^
Mean (µm)	84.2±3.5^a^	58±3.7^b^	76.7±3.0^bc^
Mode (µm)	104.6±2.6^a^	59.3±7.1^b^	83.7±2.8^c^
Nadir (µm)	61.1±1.9^a^	28.1±5.5^b^	49.7±1.8^c^
Width (µm)	16.7±0.8^a^	15.4±1.8^a^	11.6±0.4^b^
Cell number (10^6^)	10.0±1.4	10.2±2.1	11.4±2.1
**scWAT**			
Mass (g)	2.9±0.1^a^	1.2±0.1^b^	3.4±0.2^c^
Mean (µm)	61.8±4.5^ab^	53.3±2.3^a^	61.4±3.9^b^
Mode (µm)	78.1±4.2^a^	58.3±5.0^b^	83.0±2.7^a^
Nadir (µm)	46.6±3.5^a^	34.1±5.0^b^	49.4±3.7^c^
Width (µm)	14.2±2.7	14.0±1.4	13.1±2.0
Cell number (10^6^)	13.1±1.9^b^	11.8±1.2^a^	18.6±3.3^b^

Data are expressed as mean ± standard error of the mean (SEM). Different letters indicate a significant difference at p<0.05.

### Cell size distribution

**Figure 2:**
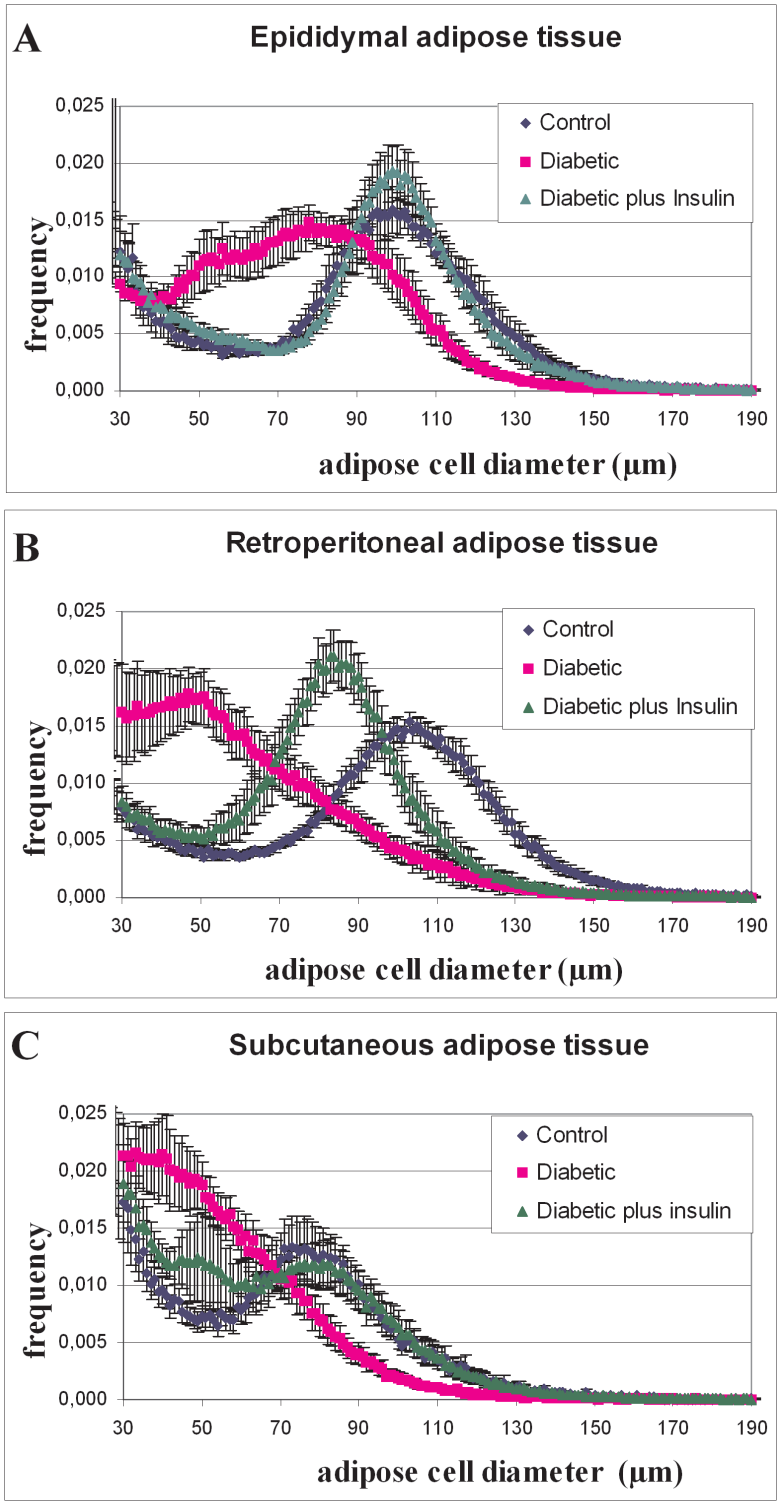
Comparison of averaged multisizer adipose cell profile curves from total adipocytes. Individual measurements were performed on 15000–21000 adipocytes from eWAT (A), rWAT (B) and scWAT (C) of control, diabetic and diabetic plus insulin rats. Frequency distribution of adipocyte sizes for controls (blue line), diabetics (red line) and diabetics supplemented in insulin (green line) treated animals. Note that the distribution of adipocyte diameters was shifted leftward (ie. toward smaller diameters) in diabetics rats. In each group, adipocytes were pooled from 7–10 rats.

As expected by the decrease in adipose tissue weights, adipose cell distributions according to their size were characterized by a shift to small cell diameters in adipose tissues of diabetic rats (**Figure 2**). The bimodal distribution of cell sizes desappeared in rWAT and scWAT of diabetic rats. The mode and the nadir were significantly decreased in all 3 adipose tissues of diabetic rats compared with the one of the control (**Table 3**). The mean was decreased only in rWAT of diabetic animals compared with the control. The width was not affected by diabetes. Insulin supplementation induced a shift to large cell size distributions, nevertheless the amplitude of that effect was different among adipose depots. Indeed, the distributions of adipose cells from epididymal and subcutaneous adipose tissues were similar in diabetic plus insulin compared to control rats. The mode returned to control values in eWAT and scWAT but not in rWAT. The nadir was significantly higher in eWAT and scWAT of diabetic plus insulin treated rats compared with the control, while it remained lower in rWAT. Interestingly, the fraction of small adipose cells, whose sizes were lower than the nadir, was significantly increased in scWAT from diabetic rats treated with insulin compared with to control (**Figure 2C**). Such a modification was not observed in either eWAT nor rWAT. In rWAT, insulin did not fully restore the distribution of adipose cell sizes to the one of the control. As can be seen in **Table 3**, the mean, the mode and the nadir measured in rWAT from diabetic rats treated with insulin did not return to control values measured in control animals.

### Cell number

From the cell size distribution it is possible to estimate cell number. Results show that cell number remained constant in eWAT and rWAT of diabetic animals. It slightly decreased in scWAT of diabetic animals. Cell number was still constant during insulin treatment in EWAT and RWAT while it returned to basal values in scWAT.

### Depot-specific basal gene expression

**Figure 3:**
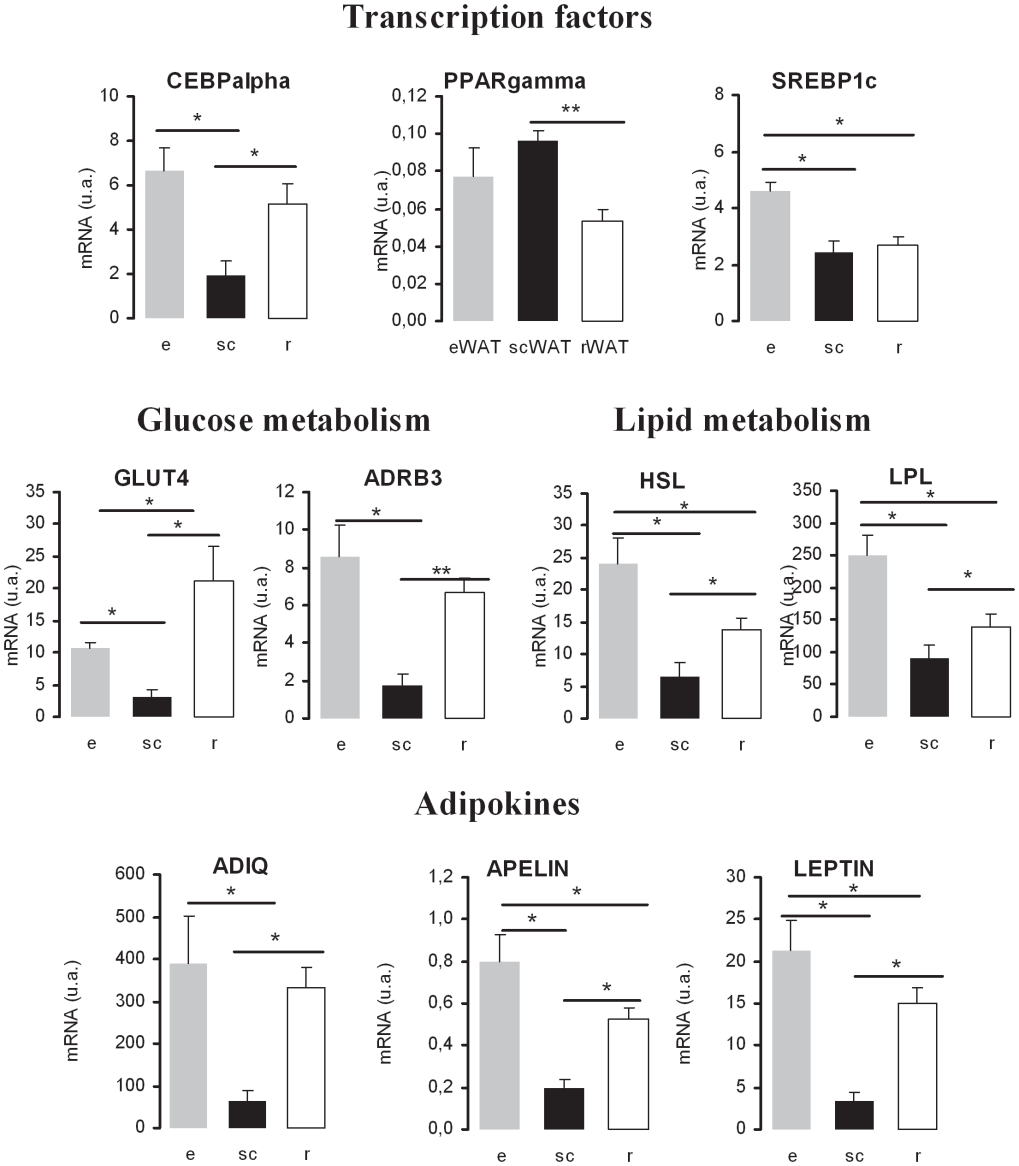
Depot-specific gene expression in rat adipose tissue. mRNA quantifications were normalized to HPRT and are represented by mean values ±SD (n = 3); ANOVA test p-values *p<0.05;. For gene nomenclature, see Table 1.

In adipocytes, gene expressions are related to their specific functions and depend both on differentiating stage and cell size. Thus, in the 3 adipose depots, we have quantified the transcripts of genes in healthy rats, representative of adipocyte maturation and main functions in both glucose and lipid metabolism, as well as adipokines and factors related to inflammation (**Table 1** **;** **Figure 3** **; Table S1**). mRNA quantifications were normalized to that of HPRT. Similar results were obtained with normalization to another standard TBP (results not shown).

Transcripts of several major transcription factors involved in adipocyte differentiation and functional maturation present different levels of expression in the 3 fat depots. Those of CEBPα were less abundant in scWAT than in eWAT. PPARγ was the only gene which transcription level was higher in scWAT than in rWAT. That of SREBP1c was higher in eWAT than in scWAT and rWAT. Transcriptional reductions in scWAT compared with eWAT and rWAT were observed for several other genes such as GLUT4, ADRβ3, *adiponectin*, *apelin* and *leptin*. The transcription level of genes coding for HSL, LPL were not significantly different between scWAT and rWAT. The only difference in gene transcription between eWAT and rWAT was for SREBP1c, which transcripts were more abundant in eWAT. Other genes coding for proteins such as FASN, Visfatin and linked to inflammatory response (TNFα, SERPINE1, MCP1, IL6) were not significantly different in basal mRNA levels within the 3 adipose tissues (**Table S1**).

### Fat depot-specific transcriptional regulation in diabetic and diabetic treated with insulin

**Figure 4:**
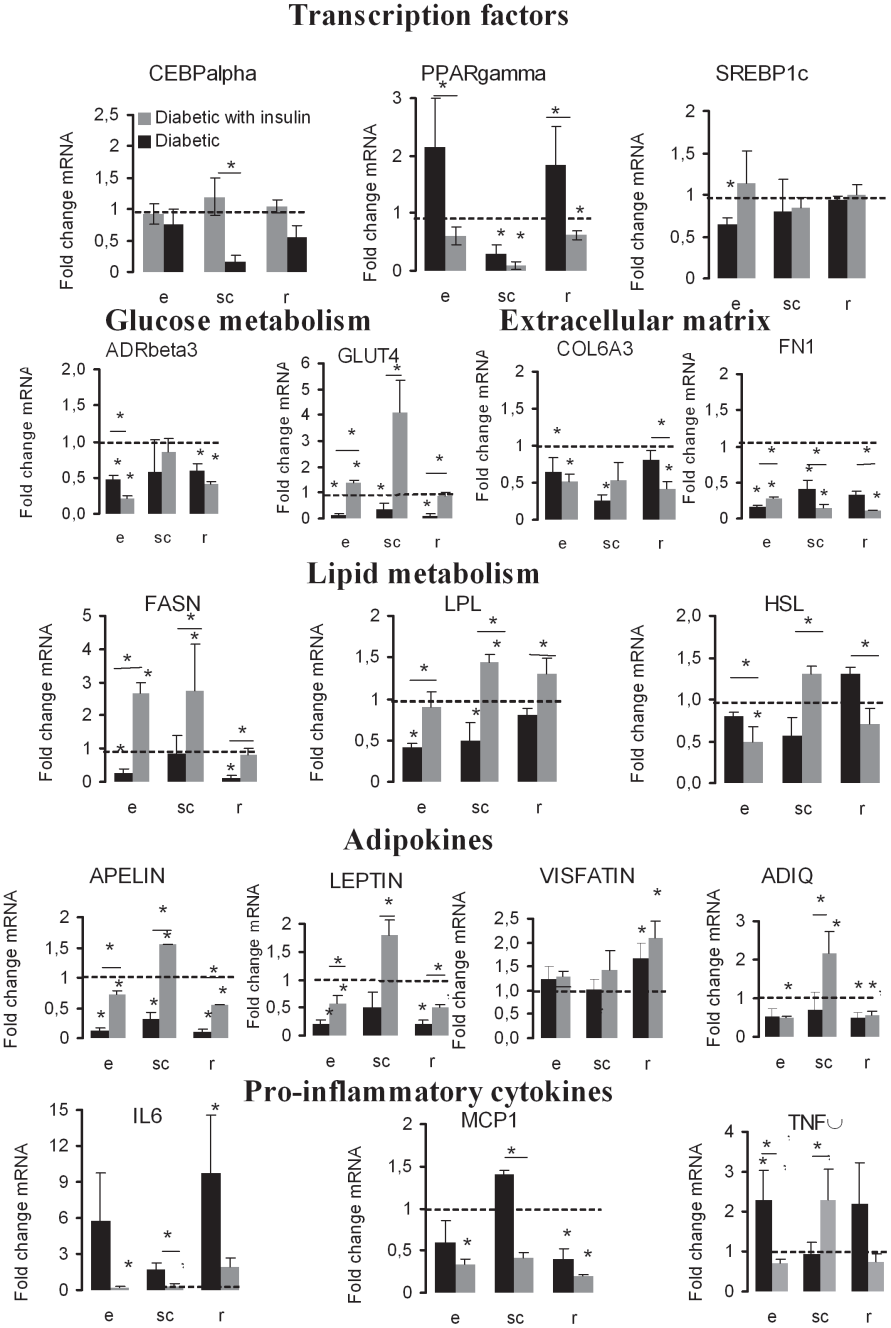
Depot specific modulation of gene expressions in diabetic and insulin-treated diabetic rats. mRNA quantifications were performed by qRT-PCR; fold changes mRNA of treated versus control rat tissues were normalized to HPRT1 and represented by mean values ±SD (n = 3) and ANOVA test-t p-values p *<0.05. For full gene nomenclature, see Table 1.

A selection of genes related to adipose tissue have been quantified by qRT-PCR in eWAT, scWAT and rWAT of untreated, diabetic and diabetic treated with insulin rats (Table S2). Genes with significant fold changes at the level of transcription are reported in Figure 4. The mRNAs coding the transcription factors CEBPα, were less abundant in diabetic animals in scWAT and rWAT than in controls, and were restored by insulin replacement in both depots. Diabetes and insulin replacement had no effect on its transcription in eWAT. The abundance of mRNA coding for PPARγ decreased only in scWAT during diabetes. Insulin replacement reduced its transcription in eWAT and rWAT but had no significant effect in scWAT. SREBP1c mRNAs were less abundant only in eWAT, not in scWAT nor in rWAT and its transcriptional level was restored by insulin replacement. The level of transcription for the receptor ADRβ3 was decreased by diabetes in eWAT and rWAT but unaltered in scWAT and a reduction of transcription level was observed in response to insulin treatment in the same depots. Diabetes significantly reduced the transcription of the gene coding for GLUT4 in eWAT and rWAT (in scWAT p = 0.585). Insulin replacement markedly increased its mRNA expression in all adipose tissues.

We further investigated whether type 1 diabetes affected the transcription of genes involved in lipid metabolism such as fatty acid synthase (FASN), lipoprotein lipase (LPL) and hormone-sensitive lipase (HSL) in each depot. FASN transcripts were less abundant in eWAT and in rWAT of diabetic animals, but significantly more abundant in response to insulin compared to diabetic animals in the three adipose tissues. LPL mRNAs were less abundant in eWAT and scWAT of diabetic animals. Insulin replacement significantly increased its transcription in the three depots. That of HSL was not altered by diabetes, was significantly reduced by insulin treatment in eWAT and rWAT but increased in scWAT.

The impact on transcriptional regulation of genes coding for adipokines was also analyzed. Adiponectin gene transcription was decreased in rWAT of diabetic rats, while it was not altered in eWAT and scWAT. Insulin replacement had no effect in eWAT and rWAT but increased its transcription in scWAT. The plasmatic concentration of the secreted protein was not altered by diabetes but increased in response to insulin replacement (**Table 2**). No difference was observed at the level of gene transcription for visfatin in eWAT and scWAT, but it increased in rWAT. Insulin replacement had no significant effect. Leptin was regulated at the transcriptional level with a significant reduction in the diabetic group in both eWAT and rWAT but not in scWAT, it was restored by insulin in eWAT and rWAT and significantly higher than the control in scWAT. Type 1 diabetes induced a down-regulation of *apelin* gene transcription in each depot, which was restored in eWAT and rWAT and up-regulated in scWAT compared to the control.

We also investigated the regulatory effect of type 1 diabetes on the transcription of genes related to inflammation such tumor necrosis factor-alpha (TNFα), interleukin-6 (IL6), monocyte chemotactic protein 1 (MCP1) and plasminogen activator inhibitor 1 (PAI1) (**Figure 4**). IL6 mRNA levels were increased in scWAT and rWAT in diabetic animals. Insulin replacement decreased its transcription, the significativity was reached only in scWAT. The mRNAs coding for MCP1 were more abundant in scWAT and less abundant in rWAT of diabetic animals. Insulin treatment reduced its transcription in the 3 depots but significant differences were found only in scWAT. The transcription of the genes coding for TNFα was increased by diabetes only in eWAT and unaltered in scWAT and rWAT, decreased in eWAT but increased in scWAT by insulin treatment, while no effect was observed in rWAT by either diabetes nor insulin replacement.

Finally we measured the transcription of several genes coding for extracellular matrix proteins (**Figure 4**). That of COL6A3 was reduced in eWAT and scWAT of diabetic animals without restoration in eWAT and even reduction in rWAT. That of FN1 was reduced in all adipose tissues of diabetic animals. It was increased in response to insulin only in eWAT but decreased in scWAT and rWAT. Under all these conditions the quantity of mRNAs remained lower than the ones measured in tissues of control animals.

### Adipocytes versus adipose tissue adipokine mRNA productions

**Figure 5:**
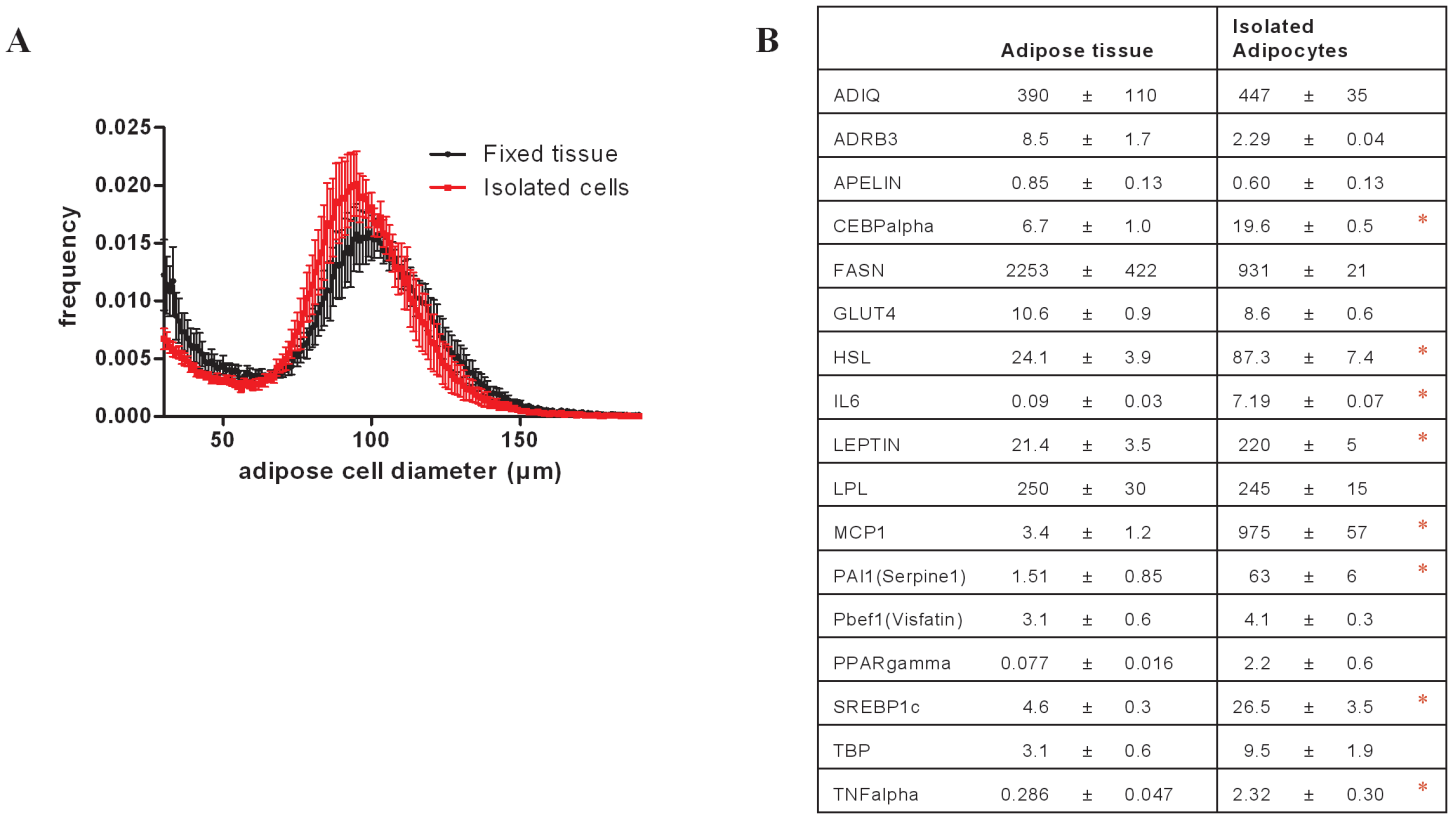
A: Comparison of averaged multisizer adipose cell profile curves from epididymal adipose tissue and from adipocytes isolated from the same tissues. B: Basal gene expression in isolated adipocytes versus corresponding epididymal adipose tissue. mRNA quantification was normalized to HPRT and is represented by mean values +/- SD, ANOVA test p-values *p<0.05. For gene nomeclature, see Table 1.

The results shown above were obtained by measuring gene rtanscriptions in the adipose tissue. Adipose tissue is a heterogeneous tissue composed of many cell types producing different adipokines. To emphasize the role of adipocytes in adipokines mRNA expressions, we compared gene expression profiles obtained from adipose tissue and from isolated adipocytes of control animals. Figure 5A shows that the cell size repartition was very similar in tissue and isolated adipose cells. The mRNA expression of several genes are very similar beween adipose tissue and adipocytes (adiponectin, ADRB3, apelin, glut 4, LPL, vistafin) suggesting that adipocytes are the main production sites for these adipokines (**Figure 5B**). Besides we found that mRNAs coding inflammatory genes were markedly increased in adipose cells compared to adipose tissue (IL6, MCP1, TNFalpha) suggesting their stimulation during the separation procedure.

## Discussion

The most important finding of the present study is that insulin deprivation profoundly altered the bimodal distribution of adipose cell sizes and insulin replacement remarkably reshaped adipose cell size distribution in each adipose tissue. Indeed, insulin deprivation not only markedly decreased adipose tissue weights (−42% in eWAT, −68% in rWAT and −58% in scWAT) but also profoundly altered adipose cell size distribution. Considering the mode, cell sizes decreased by 20% in eWAT, 31.1% in rWAT and 25.3% in scWAT. As a result, the bimodal repartition of cell size distribution was lost in rWAT and scWAT but not in eWAT of diabetic animals. The morphological changes of the adipose tissue were accompanied with changes at the level of gene transcription. For most of the genes analyzed, transcripts levels were markedly decreased in response to type 1 diabetes except PPARγ and TNFα in eWAT, MCP1 in scWAT and PPARγ, IL6 and TNFα in rWAT. Insulin deprivation decreased gene transcription of most adipokines in each fat depot except *adiponectin* and *visfatin* which were not affected in type 1 diabetes (**Figure 4**). The reduction of *leptin* gene transcription is in accordance with a markedly decreased concentration of the secreted protein in the plasma of diabetic animals (**Table 2**). Adipose tissues express leptin receptors, providing the potential for self-regulation of leptin expression, in addition to direct metabolic effects on adipocytes. Evidences from *in vitro* and *in vivo* studies suggest that the direct effects of leptin on adipocyte metabolism slightly contributes to the control of fat mass *in vivo* [27]. The absence of transcriptional regulation for *adiponectin* by insulin deprivation is also coherent with the unchanged concentration of the secreted protein in the plasma of diabetic rats compared to controls (**Table 2**).

The extracellular matrix of adipose tissue may play an important role in regulating changes in cell size. Previous studies have shown that adipose collagen VI transcripts are increased in obese subjects [28], [29] and fibronectin is known to modulate adipose cell shape and to interfere with the regulation of lipolysis [30]. After insulin deprivation, we found a reduced transcription level of both genes which can be considered as a direct effect of insulin privation on protein synthesis, thus may favor reduction in adipose cell size.

The most astonishing observation is that cell size distribution returned close to the control values in response to insulin treatment. An outstanding observation is the increased proportion of small adipose cells in scWAT after insulin treatment corresponding to a significant reduction in adipose cell size (**Figure 2C**). Estimation of adipose cell number per depot shows that cell number decreased only in scWAT in response to insulin deprivation (**Table 3**). Counting of cells by Coulter counter may not identify cells below a certain detection limit and such cells may not be detected and thus do not show up on the screen. Our results mean that adipose cell numbers have decreased but it does not necessarily mean that also the total cell number decreased. It only means that adipose cells reached size values lower than the detection limit but are most likely still present in adipose depot, as suggested by Géloën et al [20]. Our results suggest that most of the changes observed in adipose tissue result from changes in adipose cell size. This is fully coherent with recent studies using doxycycline-inducible mature adipocyte–specific tracing system in mouse, since the authors found that it is mainly adipose cell hypertrophy that explains increased adipose tissue mass in the short-term response to high fat diet [31]. Furthermore, adipocytes are fully differentiated cells that are incapable of mitotic division. This suggests that adipocyte hyperplasia arises as a result of differentiation of stem cells [32]. Although stem cell proliferation may likely occur in response to insulin replacement [20], this seems very limited since adipose cell number did not significantly increased in the different WAT depots (**Table 3**).

Although the site of insulin injection may have influenced the effects on scWAT, we do not think that the effects of insulin on scWAT are overestimated, indeed, among the 17 genes which transcripts were quantified, insulin increased the transcription of 6 of them in eWAT, 9 in scWAT and 4 in rWAT, and inhibited the transcription for 3, 2 and 6 genes, respectively. Overall, the number of genes whose transcription was altered in absence of insulin is very similar in the different adipose tissues. Whether insulin works directly at the transcriptional level or indirectly through the intermediate metabolism is an open question. SREBP1c was reported to mediate partly the transcriptional effects of insulin [33], [34], [35]. Indeed, *in vitro*, SREBP1c enhances the transcriptional activity of PPARγ, thus increasing the proportion of cells undergoing adipose differentiation. Although CEBPα has no ability to promote adipogenesis in the absence of PPARγboth CEBPα and PPARγ participate in a single pathway of fat cell development, PPARγ being the proximal effector of adipogenesis. Both transcription factors are also key regulators of lipid homeostasis [36]. The mRNA coding for PPARγ increased in intra-abdominal WAT during diabetes and decreased during insulin replacement, that is the exact opposite of adipogenesis (**Figure 4**). Early studies demonstrated that PPARγ is induced during adipogenesis and is necessary and sufficient for adipocyte differentiation [37]. One explanation of that discrepancy may lie on the fact that during deprivation and replacement, insulin only induced emptying and refilling of existing adipose cells, without inducing the differentiation of new adipocytes. *In vivo*, in normal conditions, many of the genes analyzed present a transcriptional regulation profile comparable to that of CEBPα within the 3 WAT depots (**Figure 3**). Furthermore, CEBPα transcripts were more abundant in intra-abdominal than subcutaneous WAT, and this transcriptional level is consistent with the ability of intra-abdominal adipocytes to achieve a larger size [38]. CEBPα is a transcriptional regulator of PPARγ [39], thus CEBPα may be a transcriptional regulator of adipogenesis in addition to lipid homeostasis. Whether the transcriptions of these genes are differentially regulated according to adipose cell size is elusive since they were not pointed out in the study of Jernas et al. [13] leading to identify only several genes with highly differential mRNA expressions in small versus large rat adipocytes.

With respect to the transcriptional level of transcription factors, there was no significant difference between rWAT and eWAT, except for SREBP1c which transcripts were more abundant in eWAT than in rWAT and scWAT (**Figure 3**). Most of the transcript levels were lower in scWAT than in rWAT and eWAT, except for PPARγ whose transcripts were more abundant in scWAT than in rWAT. This is especially obvious concerning the transcription of genes coding for adipokines (*adiponectin, leptin, apelin*). Compared to epididymal and retroperitoneal, subcutaneous depot was found to be less expanded, to contain smaller adipocytes, and less transcripts for GLUT4, ADRβ3, CEBPα (**Figure 3**). Plasma leptin concentration was markedly decreased by insulin deprivation and significantly increased in response to insulin replacement which is supported by results from the litterature [40]. Insulin increases leptin secretion without affecting the leptin mRNA level [41].

Several conclusions can be drawn from the effects of insulin on the distribution of adipose cell sizes. First, results show that insulin is a main regulatory factor responsible for cell size distribution in WAT. Second, since insulin exerts similarly its lipogenic effects on each adipose cell, then how is it possible to explain that the curve of cell size distribution returns back to the control? One explanation could lie on the fact that the transport of fatty acids is proportionnal to the cell surface, this is well stated for lipolysis but much less clear for lipogenesis. Besides, despite the putative surface limited lipid transport, insulin plays a critical role in lipid metabolism, by inhibiting lipolysis and promoting lipid storage in adipocytes. Insulin also stimulates the synthesis of fatty acids and triglycerides, through transcriptional induction of key lipogenic enzymes such as fatty acid synthetase (in eWAT, scAT and rWAT) and lipoprotein lipase (in eWAT, scWAT and rWAT). Whether these regulatory steps are influenced by the size of adipose cells is not yet well stated.

Expansion of fat mass mainly occurs through cellular hypertrophic mechanisms leading to an increase in adipocyte size. This has focused attention on the role of the extracellular matrix of adipose cells [42]. The extracellular matrix has been sometimes pointed out as a factor that could prevent adipocyte growth, indeed COL6A3 synthesis is increased in insulin resistant patients [29], [43]. In the present study, insulin deprivation reduced the transcription of genes coding for COL6A3, without restoration by insulin replacement but even additional reduction in rWAT. Similarly, FN1 transcript levels were reduced during type 1 diabetes and further lowered in scWAT and rWAT and only slightly increased in eWAT by insulin (**Figure 4**). These results suggest that the extracellular matrix of adipose cell is not directly involved in the changes of adipose cell sizes during highly dynamic modifications related to insulin action.

Numerous studies have shown that not only the mass of adipose tissue is important but also its quality (i.e. adipose size and size distribution) is of major importance. The present study shows that insulin is a major regulator of adipose cell size distribution and of its bimodal repartition. The mechanisms involved in that regulation are not yet fully deciphered. Understanding these mechanisms may help to identify the self organisation of adipose tissue and could bring original insights to identify new therapeutic targets for the treatment of metabolic complications induced by inappropriate fat storages.

## Supporting Information

Table S1 **qRT PCR analysis of gene transcripts in rat fat depots (epididymal: eWAT, subcutaneous; sc WAT, retroperitoneal: rWAT).** mRNA quantifications (attmol/µg) were normalized to that of HPRT. Asterix indicates ANOVA significant p-values (p<0.05).
(TIF)

Table S2 **qRT PCR analysis of gene transcripts in diabetic (D) or diabetic plus insulin (DI) rats in fat depots (epididymal eWAT, subcutaneous scWAT, retroperitoneal rWAT).** mRNA quantifications (attmol/µg) were normalized to that of HPRT. Asterix indicates ANOVA significant p-values (p<0.05).
(TIF)
