# Supplementary figures and images for: White Adipose Tissue Resilience to Insulin Deprivation and Replacement
Source: PLoS One. 2014 Aug 29;9(8):e106214. doi: 10.1371/journal.pone.0106214 (PMC4149534; doi:10.1371/journal.pone.0106214)

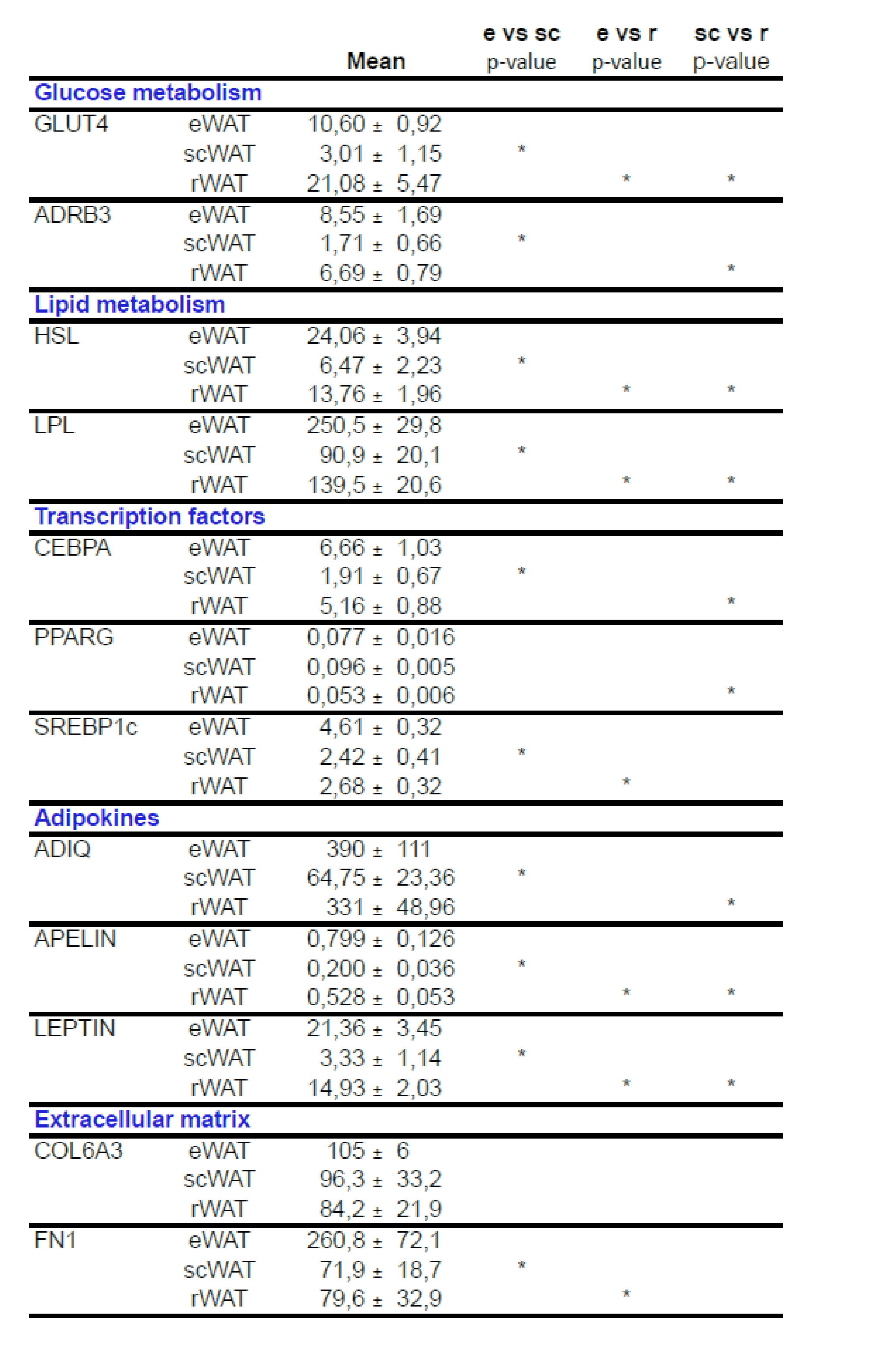

Supplement: Table S1 — qRT PCR analysis of gene transcripts in rat fat depots (epididymal: eWAT, subcutaneous; sc WAT, retroperitoneal: rWAT). mRNA quantifications (attmol/µg) were normalized to that of HPRT. Asterix indicates ANOVA significant p-values (p<0.05). (TIF) [file pone.0106214.s001.tif]

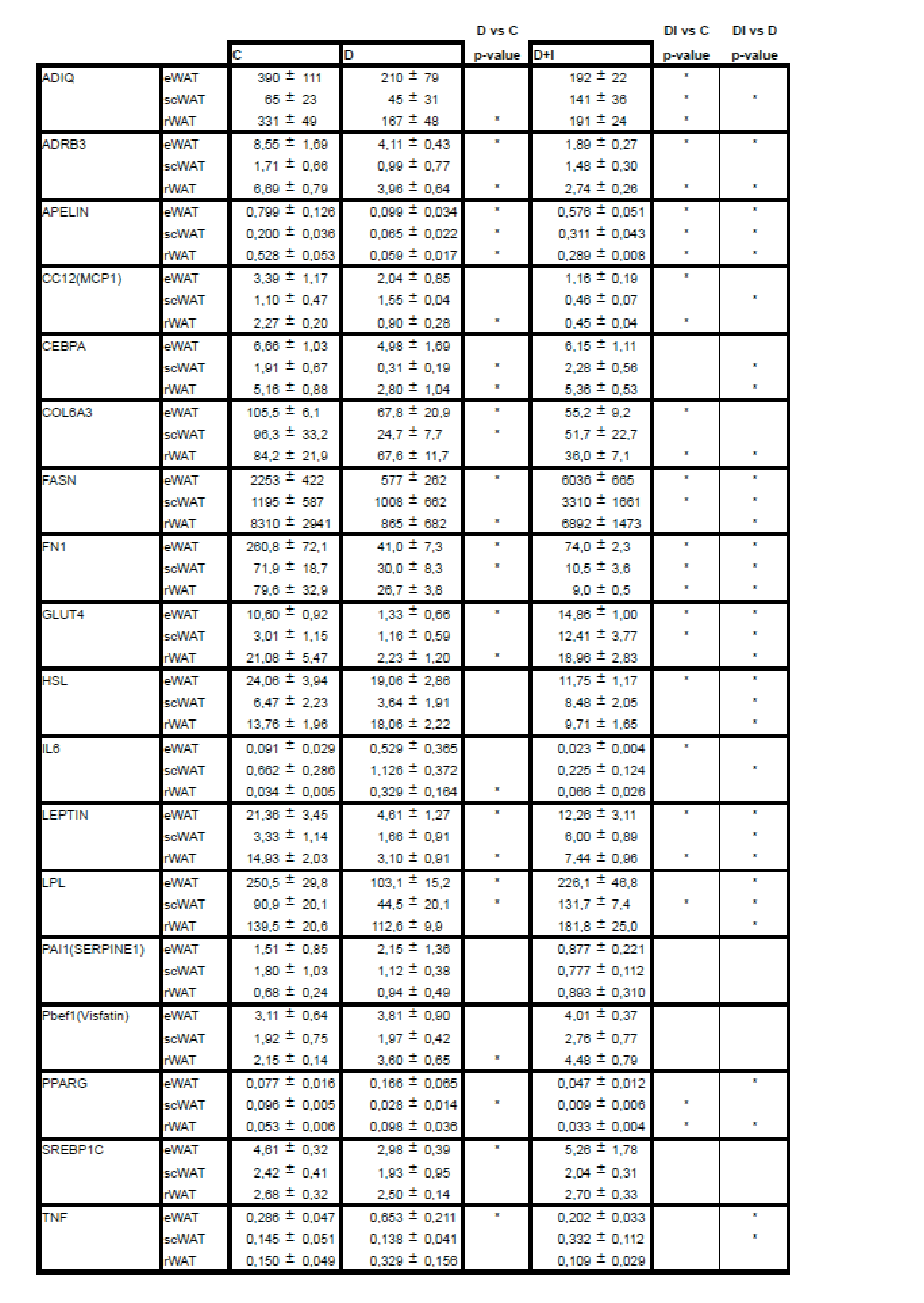

Supplement: Table S2 — qRT PCR analysis of gene transcripts in diabetic (D) or diabetic plus insulin (DI) rats in fat depots (epididymal eWAT, subcutaneous scWAT, retroperitoneal rWAT). mRNA quantifications (attmol/µg) were normalized to that of HPRT. Asterix indicates ANOVA significant p-values (p<0.05). (TIF) [file pone.0106214.s002.tif]
